# A Perfect Storm in Advanced AIDS: Severe Acute Respiratory Distress Syndrome From Pneumocystis Jirovecii and Mycobacterium Avium Complex Coinfection

**DOI:** 10.1177/23247096261475101

**Published:** 2026-07-30

**Authors:** Martinez Abdel, Rodriguez Abelardo, Conrad Chouinard, Bello Fatimah

**Affiliations:** 1 School of Medicine, 441554 The University of Texas Rio Grande Valley, Texas, USA

**Keywords:** infectious disease, pulmonary critical care, AIDS, ARDS, PJP, MAC

## Abstract

Advanced HIV infection is associated with profound immunosuppression, predisposing patients to opportunistic infections such as Pneumocystis jirovecii pneumonia (PJP) and Mycobacterium avium complex (MAC). PJP can cause severe respiratory disease, including acute respiratory distress syndrome (ARDS), while disseminated MAC may contribute to systemic illness and pulmonary complications. Concurrent infection with both pathogens is uncommon but may precipitate severe pulmonary failure. A man in his 40s presented with unintentional weight loss, fatigue, oral thrush, and progressive dyspnea. On arrival, oxygen saturation was 86% on room air, requiring high-flow nasal cannula. Laboratory evaluation revealed profound immunosuppression (CD4 7 cells/mm^3^, HIV RNA 123,000 copies/mL) and elevated β-D-glucan (>700 pg/mL). Chest CT demonstrated diffuse bilateral ground-glass opacities. He was initially treated empirically with broad-spectrum antibiotics (piperacillin-tazobactam and linezolid) and subsequently started on trimethoprim–sulfamethoxazole and corticosteroids for PJP. Despite supportive measures, the patient’s respiratory status deteriorated, requiring intubation. Sputum cultures grew MAC, prompting initiation of azithromycin, rifampin, and ethambutol. The patient developed disseminated intravascular coagulation, acute tubular necrosis requiring daily hemodialysis, and septic shock requiring dual vasopressors. He was not a candidate for ECMO due to multi-organ failure in the context of advanced AIDS, and his prognosis remained guarded. This case highlights the rapid progression and complexity of opportunistic infections in advanced AIDS. Extensive diagnostic evaluation, early recognition of co-infections, and aggressive multidisciplinary management are essential, though outcomes may remain poor in patients with profound immunosuppression and multi-organ failure.

## Background

Advanced human immunodeficiency virus (HIV) infection is associated with profound immunosuppression, particularly in patients with CD4 counts below 200 cells/mm^3^, predisposing them to a wide range of opportunistic infections.^1^ Among these, Pneumocystis jirovecii pneumonia (PJP) remains one of the most common AIDS-defining illnesses and a leading cause of severe respiratory disease in untreated or advanced HIV infection.^2^ PJP typically presents with progressive dyspnea, nonproductive cough, hypoxemia, and diffuse bilateral pulmonary infiltrates, and in severe cases may progress to acute respiratory distress syndrome (ARDS) requiring mechanical ventilation.^2,3^ Despite advances in antiretroviral therapy and prophylaxis strategies, PJP continues to be associated with significant morbidity and mortality, particularly among critically ill patients with respiratory failure.^3^

Another important opportunistic pathogen in advanced AIDS is Mycobacterium avium complex (MAC), a group of nontuberculous mycobacteria that frequently causes disseminated infection in individuals with profound immunosuppression, especially when CD4 counts fall below 50 cells/mm^3^.^4^ Disseminated MAC commonly presents with systemic manifestations including fever, weight loss, anemia, and hepatosplenomegaly, although pulmonary involvement has also been described in patients with advanced disease.^4,5^

While both PJP and MAC are well-recognized opportunistic infections in advanced HIV infection, simultaneous infection with these pathogens is uncommon and may result in severe pulmonary complications.^5,6^ Coinfections in immunocompromised hosts may complicate the clinical presentation, delay diagnosis, and contribute to worse clinical outcomes due to overlapping symptoms and the need for complex antimicrobial therapy.^6^ We present a case of severe ARDS in advanced AIDS associated with concurrent Pneumocystis jirovecii pneumonia and Mycobacterium avium complex infection, highlighting the diagnostic and therapeutic challenges in profoundly immunocompromised patients.

## Case

A man in his 40s presented to the emergency department with unintentional weight loss over the past month, worsening fatigue, oral thrush, and progressive shortness of breath over the preceding two weeks. Prior to presentation, he had been using a family member’s supplemental oxygen at home; however, his symptoms worsened to the point that he sought emergency care.

On arrival, the patient was saturating 86% on room air and required high-flow nasal cannula (HFNC) at 30 L/min with FiO_2_ of 60%. Initial laboratory evaluation revealed WBC 15.8 ×10^9^/L, hemoglobin 10.7 g/dL, hematocrit 31.7%, MCV 74.8 fL, and platelets 280 ×10^9^/L. Basic metabolic panel showed sodium 132 mmol/L, potassium 3.2 mmol/L, BUN 43 mg/dL, and creatinine 1.29 mg/dL.

Arterial blood gas on room air demonstrated pH 7.395, pCO_2_ 30.9 mmHg, pO_2_ 33.0 mmHg, and HCO_3_ 18.5 mmol/L, with a P/F ratio of 157. Given the constellation of symptoms, the patient was tested for HIV, which later returned positive.

**Figure 1.**
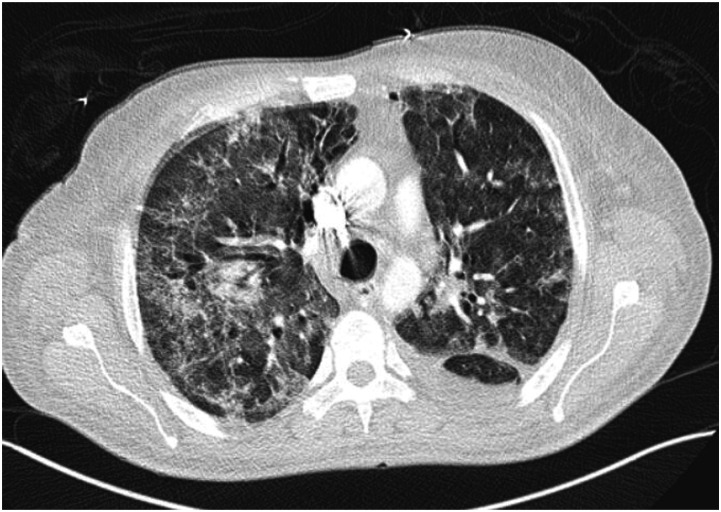
Nonspecific moderate amount of scattered groundglass pattern nonspecific pneumonitis with apparent chronic interstitial lund disease with apparent homogenous scattered cellular interstitial inflammation and suspected associated degrees of fibrosis which can be seen with nonscpecific interstitial pneumonia

**Figure 2.**
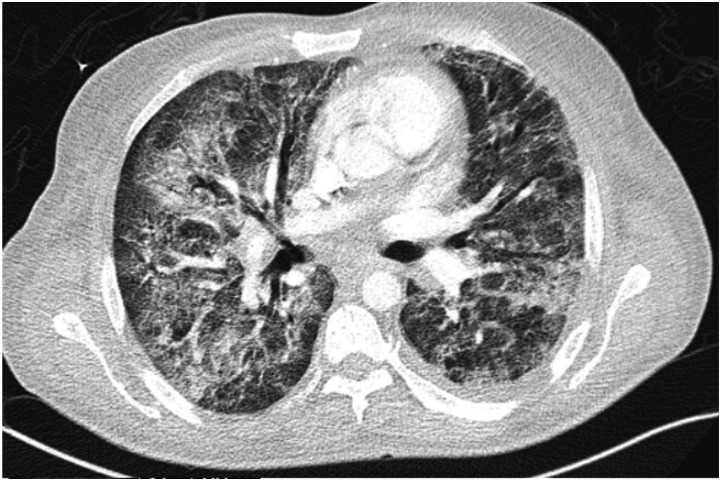
Nonspecific moderate amount of scattered groundglass pattern nonspecific pneumonitis with apparent chronic interstitial lund disease with apparent homogenous scattered cellular interstitial inflammation and suspected associated degrees of fibrosis which can be seen with nonscpecific interstitial pneumonia

Computed tomography (CT) of the chest revealed diffuse bilateral patchy ground-glass opacities (Figures 1 and 2). Pulmonology and infectious disease specialists were promptly consulted, and the patient was admitted to the intensive care unit (ICU) for acute hypoxemic respiratory failure.

Initially, the patient was managed by the admission team with empiric broad-spectrum antibiotics, including piperacillin-tazobactam and linezolid. Given the patient’s profound immunosuppression and clinical presentation, empiric treatment for Pneumocystis jirovecii pneumonia (PJP) with trimethoprim–sulfamethoxazole was subsequently initiated for a planned 21-day course, along with adjunctive corticosteroids. In addition, antifungal coverage with fluconazole was started early in the hospital course. The patient’s CD4 count was 7 cells/mm^3^ with an HIV RNA viral load of 123,000 copies/mL, and β-D-glucan levels were >700 pg/mL. Serial sputum cultures, fungal culture, PCR testing, and bronchoalveolar lavage (BAL) were obtained as part of an extensive infectious workup.

During his ICU stay, the patient initially declined intubation per advanced directives and was managed with high-flow nasal cannula (HFNC) for 5 days. Due to progressive respiratory failure, he was transitioned to noninvasive positive pressure ventilation (NPPV) with BiPAP, which he continued for an additional 9 days. Airborne isolation precautions were also implemented given imaging findings concerning for possible tuberculosis. Despite these supportive measures, his respiratory status continued to deteriorate, and after further clinical decline, the power of attorney consented to invasive mechanical ventilation, and the patient was subsequently intubated.

**Figure 3.**
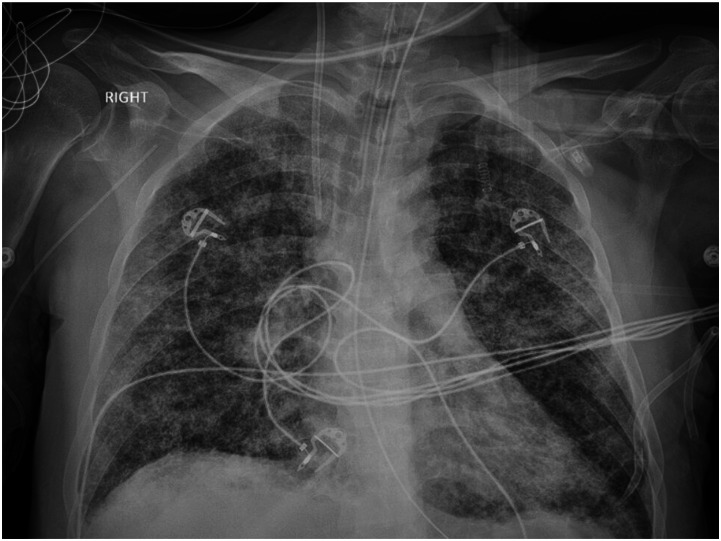
CXR demonstrates progression of diffuse interstitial infiltrates. Pneumothorax is not apparent

His clinical course progressed to acute respiratory distress syndrome (ARDS) within 7 days of the onset of worsening respiratory symptoms, fulfilling the Berlin definition timing criterion. The diagnosis of ARDS was made clinically in the setting of refractory hypoxemic respiratory failure with a severely reduced P/F ratio of 70 while on noninvasive positive pressure ventilation (BiPAP). Transthoracic echocardiography demonstrated preserved left ventricular systolic function (LVEF 50–55%) with grade II pseudonormal diastolic dysfunction, without evidence of significant acute left-sided heart failure to account for the severity of hypoxemia. The right ventricle was mildly dilated, likely reflecting increased pulmonary pressures in the setting of severe lung disease. Overall, in the context of progressive bilateral pulmonary infiltrates, worsening oxygenation despite escalating respiratory support, and absence of a primary cardiogenic etiology, the clinical picture was consistent with acute respiratory distress syndrome (ARDS) (Figure 3). Previously obtained sputum cultures returned positive for Mycobacterium avium complex (MAC), and he was started on triple antimicrobial therapy with azithromycin, rifampin, and ethambutol by the infectious disease team.

The patient subsequently developed disseminated intravascular coagulation (DIC) with worsening thrombocytopenia, necessitating discontinuation of trimethoprim–sulfamethoxazole and transition to dapsone and primaquine. Due to worsening oxygenation and arterial blood gases, he was managed according to the ARDS protocol, including dexamethasone therapy, prone positioning, and neuromuscular blockade.

The patient’s ultimate clinical outcome was poor. An extensive infectious workup, including serial sputum cultures, PCR testing, and bronchoalveolar lavage (BAL), demonstrated multidrug-resistant Enterobacter cloacae complex, Candida krusei, and Mycobacterium avium complex, leading to escalation of antimicrobial therapy to meropenem and micafungin. He also received targeted treatment for Pneumocystis jirovecii pneumonia and Mycobacterium avium complex; however, despite these interventions, he continued to clinically deteriorate. He developed refractory hypoxemic respiratory failure progressing to ARDS, septic shock requiring dual vasopressor support with norepinephrine (Levophed) and vasopressin, and disseminated intravascular coagulation. His course was further complicated by acute tubular necrosis requiring daily hemodialysis. He was not considered a candidate for extracorporeal membrane oxygenation due to multi-organ failure and poor anticipated prognosis. Despite multiple interventions, the patient died after 35 days of hospitalization.

## Conclusion

There was no prior chest CT imaging available for comparison, as the patient had no history of previous hospitalizations, and therefore no prior imaging existed to assess interval progression.

Microbiologically, the patient underwent repeated sputum cultures, PCR testing, and serial bronchoalveolar lavage (BAL), which were positive for Mycobacterium avium complex. In addition, BAL cultures grew multidrug-resistant Enterobacter cloacae complex and Candida krusei. He was also found to have an active Herpes simplex infection involving the shoulder, which was managed with valacyclovir.

Initial management included trimethoprim-sulfamethoxazole, corticosteroids, and fluconazole. Broad-spectrum antibiotics started on admission were discontinued by Infectious Diseases but later reinitiated after intubation due to concern for hospital-acquired pneumonia. Trimethoprim-sulfamethoxazole was subsequently discontinued due to concern for adverse effects, and alternative therapy with clindamycin and primaquine was initiated. Following microbiologic results as described above, antimicrobial therapy was further escalated to include micafungin and meropenem.

The patient was not receiving antiretroviral therapy at the time of hospitalization, and antiretroviral therapy was not initiated during his hospital course; therefore, immune reconstitution inflammatory syndrome (IRIS) was not a consideration.

Regarding imaging findings, serial chest radiographs demonstrated bronchiectasis and features consistent with acute respiratory distress syndrome, without evidence of cavitary lesions. A repeat CT scan was not performed during this admission, as the patient was deemed too unstable by the intensivist team—requiring paralysis, high-dose vasopressor support, and mechanical ventilation in the setting of ARDS—and additional imaging was felt unlikely to alter clinical management.

This was a highly complex case from both pulmonology and infectious diseases perspectives, with multiple potential infectious sources contributing to the clinical picture. A positive MAC culture does not necessarily establish causality. Although microbiologic data were consistently positive and treatment was initiated after multidisciplinary discussion due to worsening clinical status, the absence of classic radiographic features makes it difficult to determine whether MAC represented active infection or colonization.

This case illustrates the rapid progression and complexity of opportunistic infections in advanced AIDS, with co-infection by Pneumocystis jirovecii and Mycobacterium avium complex precipitating severe acute respiratory distress syndrome (ARDS). It underscores the importance of extensive diagnostic evaluation, including broad laboratory work-up, imaging, bronchoscopy, and testing to rule out fungal infections, to identify all potential contributors to respiratory failure.

The case demonstrates how quickly opportunistic infections can progress to respiratory failure, necessitating intubation, septic shock, and multi-organ dysfunction, highlighting the need for early recognition and aggressive multidisciplinary management. Despite adherence to evidence-based protocols, including empiric antimicrobial therapy, corticosteroids, and ARDS interventions, outcomes may remain poor in the setting of advanced immunosuppression and multi-organ failure. This underscores the necessity of timely intervention, close monitoring, and consideration of all contributing pathogens, while recognizing that even aggressive management may be insufficient in severely immunocompromised patients.
